# Blood Management: A Current Opportunity in Perioperative Medicine

**DOI:** 10.2196/57012

**Published:** 2024-03-08

**Authors:** Moises Auron

**Affiliations:** 1 Department of Hospital Medicine Cleveland Clinic Cleveland, OH United States; 2 Department of Pediatric Hospital Medicine Cleveland Clinic Children's Cleveland, OH United States; 3 Outcomes Research Consortium Cleveland, OH United States

**Keywords:** blood management, perioperative, anemia, plasma, transfusion

## Abstract

The purpose of this viewpoint is to provide awareness of the current opportunities to enhance a high-value care approach to blood product transfusion. It provides a historical context to the evolution of blood management, as well as of the patient safety and high-value care movement. Leveraging current technology for enhanced education, as well as clinical decision support, is also discussed.

## Origins of High-Value Care

The need to improve patient outcomes, with emphasis on patient safety, evidence-based decision-making, and a strong focus on high-value care, stemmed from the US Institute of Medicine’s seminal publication *To Err Is Human* [1], which was very influential in enhancing awareness of the impact of individual human behavior and decision-making on patients’ outcomes. It was a humbling and necessary perspective that spearheaded a movement toward more effective, efficient, cost-effective, and high-value–oriented practice of medicine.

## Historical Perspective on Blood Management

Blood management is not an exception to this movement. Transfusional medicine underwent tremendous development during the second half of the 20th century, faced with specific challenges such as the need for bloodless surgery in patients who refused blood transfusions and the rise of transfusion-associated viral diseases [2]. In addition, there was growing evidence of the adverse consequences associated with liberal blood transfusion, including increased mortality, sepsis, and increased length of hospitalization. This led to an awareness of the need to focus efforts on developing blood product transfusion based on the individual need of the patient, and in 2005, Isbister [3] coined the term “patient blood management.” This is a complex approach that focuses on three pillars: (1) optimizing patient hematopoiesis and enhancing red cell mass, (2) minimizing blood losses with improved source control and optimization of coagulopathy, and (3) enhancing patient tolerance to anemia [2]. In the past 30 years, substantial evidence grew to support a more restrictive transfusional approach once there was evidence that patients could tolerate lower hemoglobin values without major adverse effects; this evidence came from multiple patient populations, such as critically ill patients, older patients with high cardiovascular risk undergoing surgery, and patients with active gastrointestinal bleeding [4]. Another very important aspect that must be considered is the increasing cost associated with transfusion of blood products. Furthermore, procedures aimed to enhance patient safety (eg, pathogen reduction in platelets) substantially increase the overall cost of transfusion. A high-value care approach helps to gain insight into nontransfusional alternatives to optimize underlying hematologic conditions, but also to be cost conscious and aware of the financial impact of indiscriminate use of blood [5,6].

## Aim

The aim of this viewpoint is to allow physicians and clinicians caring for surgical patients who order blood products to reflect on the impact of the high-value care movement in blood management and transfusional medicine, as well as on the currently prevailing opportunities to enhance better decision-making; this is particularly relevant after considering the historical perspective. Ideally, the best scenario would be that patients undergo procedures and hospitalizations with minimal exposure to blood products, aiming to leverage nontransfusional correction of underlying hematologic processes. This requires enhanced awareness of current guidelines and standards of care, as well as leveraging current technology (eg, electronic health records) to help gain insight into current transfusion practices and to provide direct clinical decision-support tools that facilitate best practices in blood product ordering.

There is strong evidence of the increasing complexity of hospitalized and surgical patients [7]. It can be hypothesized that this complexity is also associated with anemia and coagulopathy as increasingly encountered comorbid conditions, especially in surgical patients. The physicians and health care professionals caring for these patients must have enhanced awareness to identify and recognize anemia and coagulopathy, with a subsequent diagnostic approach aiming to not just treat but to identify its etiology to optimize a nontransfusional approach (eg, the use of intravenous iron) [8]. A pharmacologic approach to anemia provides a more efficient and patient-centered optimization of these comorbidities with consequent enhanced treatment effectiveness and decreased adverse outcomes associated with unnecessary blood transfusion [4].

## Current Challenges

The current 2023 Association for the Advancement of Blood & Biotherapies (AABB) red blood cell transfusion guidelines have reinforced this parsimonious approach to blood transfusion, with even more conservative and restrictive levels to trigger transfusion in patients with acute coronary syndromes and pediatric patients [9]. Nonetheless, more widespread enhanced adherence to the AABB guidelines in regard to red blood cell transfusion is a necessity. In addition, plasma transfusion offers a strong opportunity for improvement in health care delivery, especially as there is a need to minimize unnecessary plasma transfusion as well as its inappropriate dosing; plasma should be transfused with weight-based dosing and in appropriate clinical scenarios. Undertransfusion of plasma, by not using weight-based dosing, is a current challenge as this not only does not have a therapeutic corrective effect on coagulopathy but is a source of wastage [10]. Enhanced education efforts worldwide, as well as leverage of current technology, create awareness and encourage adoption of a high-value approach to plasma and red blood cell transfusion. Another element to consider as a balancing measure to enhanced patient safety is the increased associated cost; in the case of platelet transfusion, in the United States the current standard of care is the use of pathogen-reduced platelets; this approach increases costs of individual blood products substantially [6].

The perioperative continuum of care provides different stages to ensure that patients are properly evaluated and treated. In the preoperative setting, the optimization of anemia carries the most significant value through raising hemoglobin values to levels high enough to minimize reaching the transfusion threshold while also enhancing overall oxygen delivery [4]. In the intraoperative setting, the leverage of cell-saver technology, as well as optimization of coagulopathy, can mitigate the risk of blood product use; however, awareness of appropriate indications as well as of dosing of blood products promotes a high-value approach and minimizes wastage [9,10]. In the postoperative realm, it involves ensuring appropriate monitoring of ongoing blood losses, as well as monitoring the patient for potential complications associated with postsurgical anemia, such as myocardial ischemia in noncardiac surgery [11].

## Potential Solutions and Opportunities

What can be done to mitigate the inappropriate overuse of blood products, inappropriate dosing, and lack of awareness of the associated costs? Appropriate data bank analysis and data-driven interventions, as well as the implementation of human factors engineering and newer technologies such as artificial intelligence within the current workflow (like the electronic health record), can enhance the effectiveness of patient blood management efforts [12]. This entails having a database of all patients being transfused in a hospital or health care system and being able to have granularity to drill down to data on the individual patient, ordering physician, and baseline and posttransfusion laboratory values (eg, complete blood count), as well as associated outcome metrics like readmissions, length of stay, and cost of care. In addition, short-cycle data, which allow immediate identification of patients who can benefit from further stratification and assessment of underlying anemia and coagulopathy, permits guiding clinicians to pursue real-time high-value care and evidence-based interventions supported by clinical decision support tools. Also, data governance of anemia and coagulopathy assessment, as well as blood transfusion practices, provides a platform for permit auditing, benchmarking best practices, and providing real-time feedback to individual physicians, increasing awareness of areas of success and opportunities [13].

The electronic health record also provides a strong platform for education, as clinical decision-support tools can be embedded in the orders [14]. For instance, in our institution, we default red blood cell transfusion orders to single units and have a formal indication: What is the current transfusion threshold? This allows the ordering health care professional to reflect and select a reason when the order does not follow the current AABB guidelines. Also, when plasma is ordered, there is an indication to use weight-based volumes to minimize undertransfusion, as well as education that transfusion for an international normalized ratio <1.8 will not have a meaningful impact. Order overriding can occur, but with the need to provide a rationale. The more the orders are used and experience increases with blood product transfusion, the more exposure there will be to this workflow, allowing for enhanced education. Also, the electronic health record can facilitate improved documentation of blood product transfusions, allowing the development of increased insight into potential blood product overuse [15].

In this issue of *JMIR Perioperative Medicine*, we provide the opportunity to outline the evidence for evaluation and optimization of perioperative anemia in different surgical populations, as well as to discuss the opportunities for leverage of current technologies to enhance the effectiveness of approaches to improve patient outcomes and enhance the high-value care approach, minimizing not only financial costs, but more importantly, decreasing patient harm.

## Abbreviations

AABB: Association for the Advancement of Blood & Biotherapies
